# Demonstrating Feasibility of Point of Care Ultrasound (POCUS)-Guided Inpatient Transthoracic Echo Triage Decision Pathway

**DOI:** 10.24908/pocusj.v10i01.17776

**Published:** 2025-04-15

**Authors:** Stephanie M. Conner, Mustafa Husaini, Maya Fiore, Mohamed Ramadan, Benjamin Hoemann, Nicholas Arnold, Farhan Katchi, Crystal Atwood, Carol Faulk, Karl Wallenkampf, Jing Li

**Affiliations:** 1 Washington University in St. Louis, Department of Medicine, Division of Hospital Medicine, St. Louis, MO, USA; 2 Washington University in St. Louis, Department of Medicine, Division of Cardiology, St. Louis, MO, USA; 3 Washington University in St. Louis, Department of Medicine, Division of General Medicine Sciences, St. Louis, MO, USA; 4 Columbia University Irving Medical Center, Department of Medicine, Division of General Medicine, New York, NY, USA; 5 University of South Carolina School of Medicine Greenville, Columbia, SC, USA; 6 Marshall University, Department of Pulmonary and Critical Care, Huntington, WV, USA

**Keywords:** cardiac POCUS, echocardiography, quality improvement, length-of-stay

## Abstract

**Background:**

Prolonged inpatient length of stay (LOS) is associated with worse clinical outcomes and increased healthcare costs. Transthoracic echocardiography (TTE) is commonly utilized in cardiac evaluation of hospital inpatients but is associated with prolonged LOS and may not always be necessary. Point of care ultrasound (POCUS) may help reduce the need for inpatient TTEs.

**Objective:**

We aimed to demonstrate the feasibility of a POCUS-guided TTE triage protocol and estimate its impact on inpatient TTE utilization.

**Methods:**

From September to December 2023, inpatient clinicians and participating patients at a large academic institution were surveyed about their perspectives and experiences with POCUS. Cardiac POCUS exams were performed and interpreted for pre-specified clinical indications by POCUS-trained hospitalists, then reviewed independently by at least two board-certified cardiologists. Interpretations were compared using pairwise agreement analysis (kappa (κ) statistic). Finally, hospitalists and cardiologists independently offered their TTE triage recommendation, categorized as either inpatient, outpatient, or cancellation. Triage agreement between the two groups was reported as a percentage of overall cases.

**Results:**

Clinicians and patients were receptive to integrating POCUS exams into clinical care. Ninety POCUS exams were completed during the intervention period, on average 22 hours before TTE. Hospitalist and cardiologist agreement was moderate to very good (0.57-0.99) for specific cardiac findings. The hospitalist and at least one cardiologist agreed that 59 (66%) of 90 exams performed within the triage pathway could result in deferral or cancellation of inpatient TTE.

**Conclusions:**

A POCUS-guided TTE triage protocol can reduce low-value inpatient TTE use, potentially expediting necessary TTEs and reducing TTE backlog.

## Background

Prolonged length of stay (LOS) results in increased healthcare costs and decreased bed availability [1,2]. For patients, longer LOS is associated with increased rates of depression, mortality, hospital-acquired infections, and loss of mobility [3]. Delays in diagnostic testing are known to contribute to prolonged LOS. A transthoracic echocardiogram (TTE) is a useful clinical tool for cardiac evaluation of hospitalized patients. However, it has been associated with significant delays to complete and has been associated with prolonged inpatient LOS [4]. TTE use doubled amongst Medicare beneficiaries from 1999 to 2008, and in 2019 over 7.5 million TTEs were performed [5,6]. Guideline recommendations for ordering an inpatient TTE vary from high-quality evidence (e.g., re-evaluation of known or suspected heart failure (HF)) to low quality (e.g., establishing diagnosis of suspected pulmonary embolism), and practice patterns between and within institutions can vary significantly [7]. Efforts to reduce LOS by reducing unnecessary inpatient TTE orders (and other radiologic studies) are an area of active research. One successful algorithm used brain natriuretic peptide (BNP) to triage TTE orders, which modestly reduced inpatient TTE orders in patients with HF with no increased adverse events [8].

Point of care ultrasound (POCUS) is an attractive alternative to a biomarker-based algorithm for several reasons. Prior studies have demonstrated that POCUS-trained providers can safely perform and interpret cardiac POCUS exams with non-inferiority to board-certified cardiologists for specific clinical indications. These include assessment of left ventricular (LV) systolic function, pericardial effusion, right ventricular (RV) size and function, and interpretation of the inferior vena cava (IVC) [9–11]. Additionally, POCUS improves diagnostic accuracy and can expedite diagnosis for certain cardiopulmonary conditions, which makes it potentially useful in triaging the necessity of inpatient TTE [12–14]. Recently, the American Society of Echocardiography and the American College of Cardiology have released working documents aiming to better define the potential utility of POCUS to augment—or spare—TTE use [15,16].

At our institution, inpatient TTE requests exceeded the capacity of the Cardiac Diagnostic Laboratory to perform them within the ideal timeframe (24 hours or less). From January to October 2023, the average time from TTE order to report finalization on the inpatient Internal Medicine service was 31.1 hours, with significant variability between mid-week and weekends. About half of TTEs took more than 24 hours to complete.

In this study, we developed and tested a POCUS-guided TTE triage protocol to demonstrate its feasibility and estimate impact on inpatient TTE utilization. We aimed to (1) assess clinician and patient receptivity to POCUS use, (2) demonstrate that POCUS exams can be performed reliably and in a timely manner by POCUS-trained hospitalists for specific cardiac findings, and (3) evaluate potential impact on inpatient TTE use as a result of the pathway.

## Methods

### Study Setting

This study was conducted at a large, urban, tertiary referral hospital between September and December 2023. The Washington University School of Medicine Institutional Review Board (IRB) reviewed this study and determined that it qualified as a quality improvement project, meaning IRB oversight was not required.

### POCUS-guided TTE Triage Pathway

**Figure 1.**
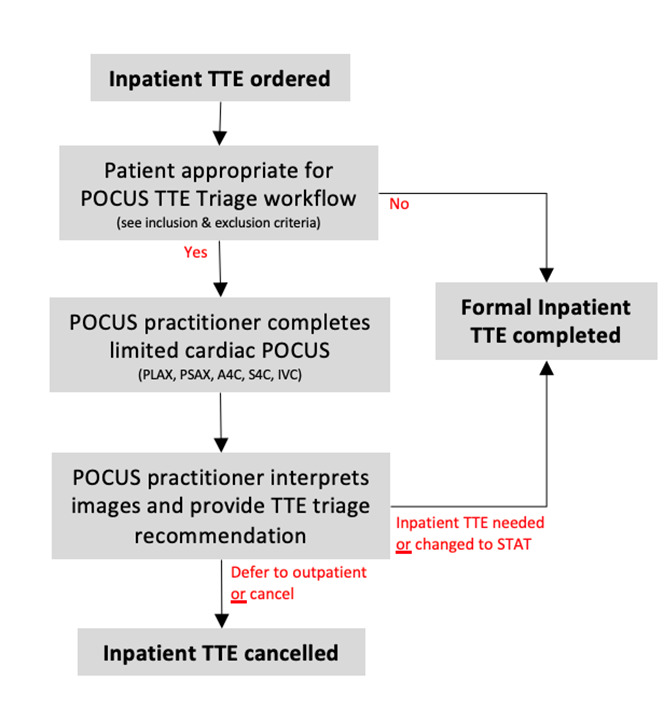
Point of care ultrasound (POCUS)-guided transthoracic echocardiography (TTE) triage algorithm. Existing TTE orders are screened for appropriateness using inclusion and exclusion criteria, then referred to POCUS hospitalists if appropriate. TTE triage (continue with inpatient TTE vs. cancel or defer to outpatient) is dependent on the POCUS practitioner's exam quality, interpretation of findings, and clinical context.

The POCUS-guided TTE triage decision pathway (Figure 1), inclusion criteria, and exclusion criteria were developed by negotiated consensus between stakeholders from both Hospital Medicine and Cardiology. These incorporated knowledge of frequent TTE order indications as well as where POCUS might be most useful given its limited clinical applications. A research team member screened the daily TTE worklist available on the Electronic Health Record using a convenience sample approach, then referred patients who met inclusion and exclusion criteria to a POCUS hospitalist for chart review and examination. In rare cases, the POCUS hospitalist could self-identify an appropriate patient for inclusion from their clinically assigned patient list.

Eligible patients included adults (>18-years-old) admitted to the Medicine or Cardiology acute care medical wards (non-Intensive Care Unit) with an inpatient TTE order placed by the primary clinician or admitting team for one or more of the following indications: decompensated heart failure with reduced ejection fraction (HFrEF) with recent TTE (<3 months), isolated BNP elevation, dyspnea with alternative clinical explanation, lower extremity edema with alternative clinical explanation, low risk chest pain or acute coronary syndrome, or non-cardiac admitting diagnosis. Patients were excluded if they had one or more of the following: known mechanical or prosthetic valve, known congenital heart disease, known left ventricular assist device, or strong clinical indication for comprehensive inpatient TTE (e.g., pre-chemotherapy, pre/post cardiothoracic surgery, stroke evaluation, or endocarditis evaluation).

A cardiac POCUS exam was performed, including parasternal long axis, parasternal short axis, apical 4-chamber, subcostal 4-chamber, and longitudinal IVC view as able by the POCUS hospitalist. Two dimensional images were obtained for all patients with M-mode and color Doppler utilized per hospitalist level of training. All images were obtained using either a Sonosite PX cart-based ultrasound (Bothwell, WA) or Philips Lumify ultraportable ultrasound (Amsterdam, Netherlands), stored locally on the respective device, and recorded using a random identification generator to avoid including protected health information. Images were uploaded to a secure online platform for further processing and review (Box; Redwood City, CA).

### Aim #1: Clinician and patient receptivity to POCUS use

Clinician receptivity to POCUS was assessed as part of a 14-question online survey using Qualtrics (Seattle, WA) and was disseminated via email to all Internal Medicine residents (postgraduate year (PGY)-1, PGY-2, and PGY-3), hospitalists (physicians and advanced practice clinicians), inpatient-practicing cardiologists (attendings, fellows, and advanced practice clinicians), and inpatient-practicing Internal Medicine and Subspecialty attendings. The survey was developed based on the study's objectives, team members' expertise, and relevant literature. Two study team members drafted the initial questions, followed by an iterative review process that incorporated feedback and pilot testing from the extended team for content and face validity. This survey queried basic demographic and practice information and used a 5-point Likert scale to assess overall experience with inpatient diagnostic testing and perceived quality of care, and perspectives of POCUS. Additionally, a brief description of the proposed POCUS-guided TTE triage pathway was given, and clinicians were asked to rate their comfort using or following certified POCUS users' recommendations with the pathway. Responses to Likert scale questions were analyzed by clinician group (residents, hospitalists, cardiologists, Internal Medicine/subspecialty) using a t-test in R (Indianapolis, IN) and reported as an average and standard deviation.

Patient experience with POCUS was assessed as part of a 22-item voluntary online survey in Redcap (Nashville, TN) within 24 hours of having a POCUS exam completed as part of the TTE triage pathway. This survey queried basic demographic information, understanding of illness, and perspectives around efficiency and quality of inpatient care, particularly related to diagnostic testing and communication with their clinical teams. Additionally, patients were specifically asked about several variables related to the POCUS exam, including discomfort or inconvenience related to the exam, perceived clinician knowledge, contribution to understanding of illness, and overall patient experience. Patient responses to POCUS-specific questions were aggregated and reported as average and standard deviation for each question.

### Aim #2: Hospitalist POCUS timeliness and reliability

POCUS exams were performed and interpreted by six POCUS-trained hospitalists and Internal Medicine residents. Two hospitalists (SMC, MR) had previously completed a national society-sponsored POCUS Certificate of Completion; the remaining four were in the process of a similar Certificate of Completion and/or had completed more than 20 hours of prior instruction.

POCUS-trained hospitalists completed a clinical questionnaire using online survey software in Redcap (Nashville, TN) including exam identification, medical record number, date of exam, obtained views (cardiac and other), brief clinical history, and indication for TTE. The hospitalist self-assessed overall image quality and offered an interpretation of LV systolic function, LV size, RV size and function, IVC size and collapsibility, and pericardial effusion. Finally, they offered a TTE triage recommendation, with optional free-text comments: change TTE order to STAT, keep inpatient TTE order, defer TTE to outpatient, or cancel TTE.

A member of the research team (MF) collated the brief clinical history, indication for TTE, and recorded images into a presentation for blinded review by at least two of the three participating cardiologists, all board-certified by the National Board of Echocardiography (Raleigh, NC). Each cardiologist independently reviewed the images for overall image quality, interpretation of the POCUS findings, and TTE triage recommendation.

The timing of the POCUS exam was recorded by exam timestamp. POCUS-performed time to TTE Electronic Health Record order time reported in Epic, (Verona, WI), was recorded as time in hours and standard deviation. Any POCUS exams that were performed before TTE order were excluded so as not to falsely reduce observed timing. TTE completion time to TTE order time was reported similarly. No statistical analysis was performed.

The reliability of hospitalist POCUS image interpretation was assessed by comparing to cardiologist interpretation. To facilitate a pairwise agreement analysis, each interpreted variable was designated a binary outcome. For instance, “0” was indicated if left ventricular systolic function (LVSF) was interpreted as normal or hyperdynamic and “1” was indicated if reduced or severely reduced. If the hospitalist or cardiologist selected “unable to assess” or “not assessed,” no outcome was applied. See Appendix 1 for full details of outcomes by variable. Additionally, discordant outcomes between cardiologists were resolved either by acceptance of an outcome if no alternative outcome was offered, or by a third cardiologist independent review for tie-breaking. The κ statistic and associated p-value were generated for each POCUS variable, using the following ranges for interpretation [17]:

- *< 0 indicates less than chance agreement,*
- *0–0.20 represents slight agreement,*
- *0.21–0.40 represents fair agreement,*
- *0.41–0.60 represents moderate agreement,*
- *0.61–0.80 represents substantial agreement, and*
- *0.81–1 represents almost perfect agreement.*

Sample size was calculated based on disagreement probability, Type I error, and Type II error. With a Type I error of 0.05 and a Type II error of 0.2 (80% power), the minimum required sample size was 81 for a 30% disagreement probability and 96 for a 50% disagreement probability.

### Aim #3: Potential impact on inpatient TTE use

TTE triage decision was recorded for each patient by the performing hospitalist and both cardiologists. As above, TTE triage decisions were designated into binary outcomes; “0” if TTE was recommended to be deferred to outpatient or cancelled altogether, or “1” if recommended to remain inpatient or to be escalated to STAT. After discussion by the research team and consideration of the lack of strong guidelines for inpatient TTE use for the indications included within the study, the TTE triage decision was not analyzed by pairwise agreement analysis as this was deemed to oversimplify the complexity of triage decision-making. Instead, TTE triage decisions were grouped by hospitalist and cardiologist agreement for deferral/cancellation (H-C-), inpatient/STAT (H+C+), or hospitalist and cardiologist discordance (H-C+, H+C-), and reported as a percentage of overall cases. Agreement was defined as the agreement between a hospitalist and at least one cardiologist; the rates of single cardiologist vs. both cardiologist agreement are also reported.

## Results

### Aim #1: Clinician and patient receptivity to POCUS use

Of 463 clinicians contacted, 153 (31%) completed the survey, including 49/154 (32%) residents, 61/130 (47%) hospitalists, 32/128 (25%) cardiologists, and 11/51 (21%) Internal Medicine/subspecialty attendings. Overall, clinicians had a neutral-to-negative opinion regarding the timeliness of patient care in the hospital (2.87 ± 1.04), particularly regarding diagnostic testing (2.83 ± 0.97). As shown in Table 1, all groups except cardiologists expressed some discomfort with a TTE triage protocol that would require them to personally perform POCUS; this significantly improved when offered for a certified POCUS user to complete the exam and provide a recommendation.

**Table 1.** Shows 5-point Likert scale survey responses (1 = very uncomfortable; 5 = very comfortable) from hospitalists (n = 61), cardiologists (n = 32), Internal Medicine trainees (n = 49), and outpatient or subspecialty Internal Medicine attendings on resident teaching services (n = 11) comparing perspectives personally using vs. following certified point of care ultrasound (POCUS) users' recommendations on the described transthoracic echocardiography (TTE) triage pathway.

Clinician group	I would feel comfortable ***personally using*** POCUS to triage inpatient TTE orders.	I would feel comfortable ***following certified POCUS users' recommendations*** to triage inpatient TTE orders.	p-value (t-test)
Hospitalists	2.35 (1.21)	3.85 (0.79)	**<0.01**
Cardiologists	3.52 (1.29)	3.03 (1.30)	0.15
Trainees	2.59 (1.00)	3.68 (0.82)	**<0.01**
Other Attendings	2.63 (1.41)	3.44 (1.03)	0.07

**Figure 2.**
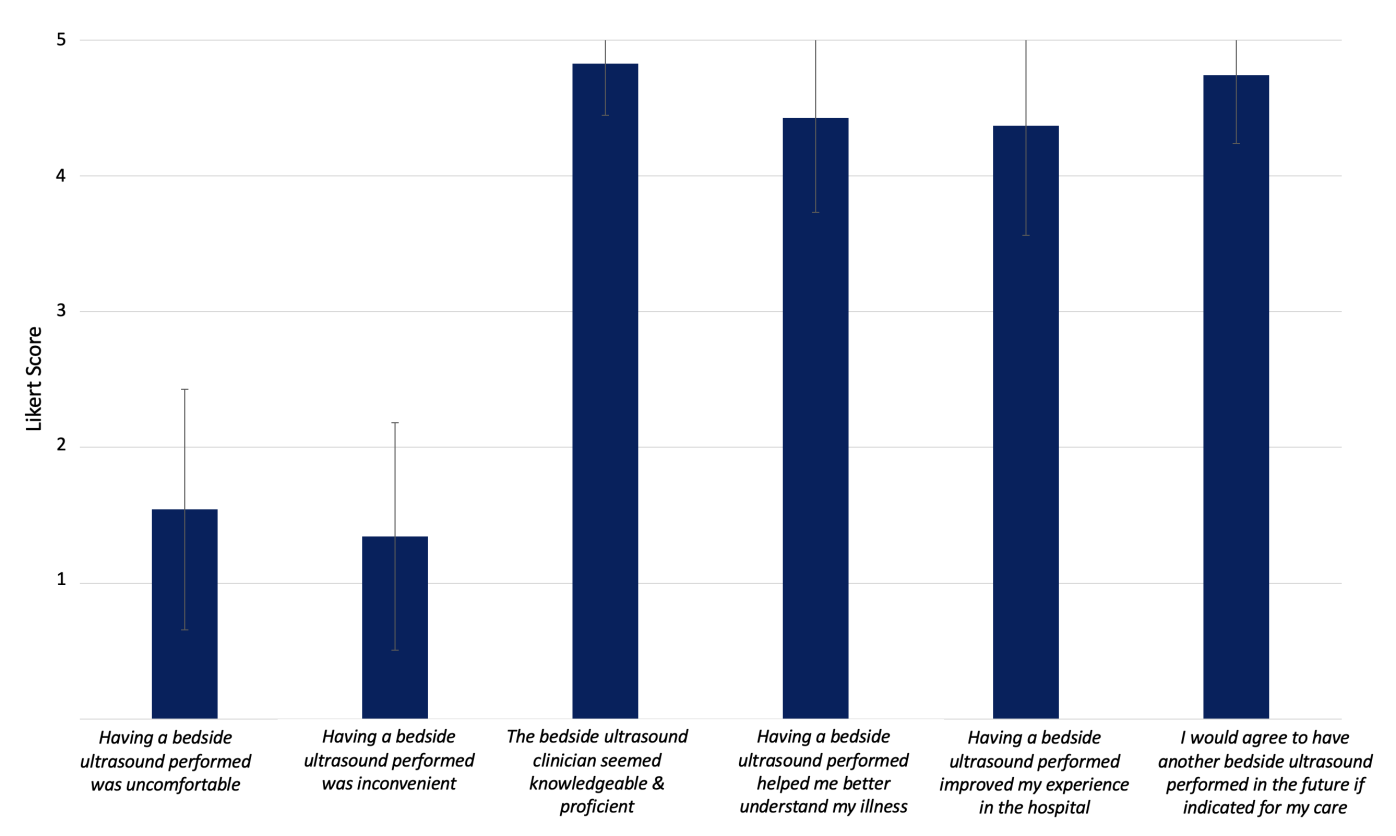
Results of patient survey responses to experience with point of care ultrasound (POCUS) exam (n=35), reported as average ± SD. Responses were recorded according to standard 5-point Likert scale from strongly disagree (1) to strongly agree (5).

Ninety patients were included in the POCUS-guided TTE triage pathway; of these, 35 consented to complete the survey (39%). Patient demographic variables of the surveyed and unsurveyed group are provided in Appendix 2. Notably, significantly more female patients were amenable to voluntary survey (60% vs. 29%, p = 0.02), though no other significant difference between groups was seen. Figure 2 shows patient responses to POCUS-specific questions. Importantly, patients did not report significant discomfort (1.54 ± 0.89) or inconvenience with having a POCUS exam performed (1.34 ± 0.84); on the contrary, patients noted an improved sense of illness understanding (4.43 ± 0.70) and overall inpatient experience (4.37 ± 0.81) after having the exam performed.

### Aim #2: Hospitalist POCUS timeliness and reliability

On average, POCUS exams were completed 21.3 ± 19.0 hours from TTE order; TTE was completed in 43.3 ± 30.0 hours. Thirteen of 90 (14%) POCUS exams were completed before TTE was ordered and excluded from timing analysis. Additionally, 14 (15.5%) TTE orders were cancelled by the primary clinical team after the initial order or not completed by the time of discharge and were not available for subsequent analysis. Of the 90 studies performed, 84 (93%) were deemed “fair” or “good” quality for at least limited interpretation.

Hospitalist and cardiologist agreement on POCUS findings is summarized in Table 2. Using previously described definitions [17], agreement was almost perfect for LVSF (0.84, 0.71-0.98), substantial for RV size/function (0.66, 0.43-0.89), and moderate for LV size (0.57, 0.36-0.79). Because our study sample lacked abnormal pericardial effusion studies, the κ statistic could not be measured. However, the negative agreement between hospitalist and cardiologist was almost perfect (0.99, 0.98-0.99).

**Table 2.** Results of pairwise agreement analysis between hospitalist and cardiologist interpretation for each point of care ultrasound (POCUS) finding after binary classification.

POCUS Variable	κ (95% CI)	p-value
LV size	0.57 (0.36, 0.79)	**<0.001**
Pericardial effusion	0.99 (0.98, 0.99)	**<0.001**
RV size/function	0.66 (0.43, 0.89)	**<0.001**
LVSF	0.84 (0.71, 0.,98)	**<0.001**

Agreement on pericardial effusion and left ventricular systolic function (LVSF) were “almost perfect”, right ventricular (RV) size/function was “substantial,” and left ventricular (LV) size was “moderate”. *Note that pericardial effusion agreement was calculated as positive agreement (instead of overall) given lack of abnormal (1) codes for this finding in the dataset.

### Aim #3: Potential impact on inpatient TTE use

**Figure 3.**
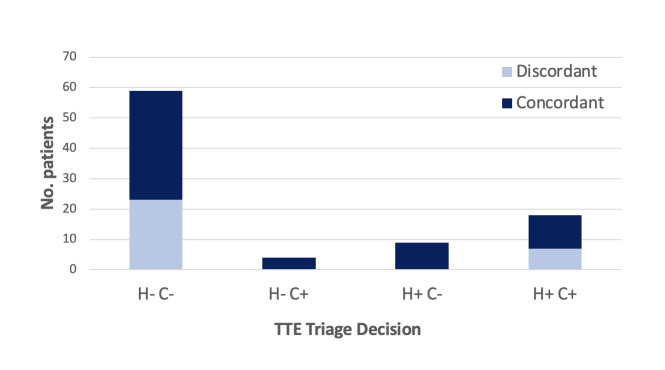
Results of transthoracic echocardiography (TTE) triage recommendation by hospitalist (H) and cardiologist (C), reported as number of patients per triage decision. Discordance between cardiologists (light blue) is differentiated from full concordance between hospitalist and both cardiologists (dark blue). The most common occurrence was hospitalist and cardiologist recommended TTE deferral to outpatient or cancellation (H-C-, n=59)

Of the 90 exams performed, the hospitalist and at least one cardiologist agreed that 59 (66%) of inpatient TTEs captured by the POCUS-guided triage pathway could be deferred or cancelled (Figure 3). More than half of these (36/59, 61%) were fully concordant between hospitalist and both cardiologists. There were four cases where both cardiologists agreed that a TTE should occur inpatient and the hospitalist had elected to defer or cancel. Of these cases, two were cancelled by the primary clinical team before inpatient completion (not related to the triage pathway) and the remaining two had no significant findings on review of TTE.

## Discussion

This study demonstrated the feasibility of a POCUS-guided TTE triage protocol with the potential to reduce inpatient TTE utilization. We demonstrated that clinicians and patients are receptive to POCUS utilization, trained hospitalists can reliably and timely perform POCUS exams for specific cardiac findings, and that the described protocol could meaningfully reduce unnecessary inpatient TTE use at our institution.

Buy-in is a cornerstone to many change management, dissemination and implementation efforts [18,19]. As a result, obtaining clinician and patient buy-in for integrating POCUS into clinical decision-making is a crucial first step to the success of this pathway. Results of the clinician survey suggested that clinicians are receptive to POCUS use by certified users for this purpose, and patient surveys revealed positive experiences with POCUS in general and performing clinicians. As expected, cardiologists were more comfortable self-performing a POCUS exam than general practice clinicians (trainees, hospitalists, and non-cardiologist subspecialty teaching attendings). Increasing the availability of POCUS training, continuing to emphasize minimum required experience, and making equipment more accessible to front-line clinicians will narrow this gap. Over time, this can further increase the relevance and impact of such POCUS-guided triage algorithms.

The favorable agreement analysis between hospitalist and cardiologist image interpretation supports previous literature that suggests trained POCUS users can reliably perform and interpret certain qualitative findings from cardiac ultrasound. There was variability in agreement between hospitalist and cardiologist interpretation of findings, with “almost perfect” agreement on LVSF and pericardial effusion, “substantial” agreement on RV size/function, and “moderate” agreement on LV size. This reflects the relative complexity of RV and LV size assessment compared to LVSF and pericardial effusion from basic cardiac POCUS views. Comprehensive echocardiograms, in contrast, include numerous, redundant views designed to minimize the impact of artifacts, foreshortening, or suboptimal cross-sectional imaging planes. In contrast, POCUS is inherently limited to fewer views, which increases the potential for variability in image acquisition and interpretation.

The additional benefit of timeliness compounds the potential for POCUS to expedite appropriate TTE utilization and reduce waste from inappropriate or low-value TTEs. This study contributes to a growing body of literature showing that incorporating POCUS into clinical decision-making can impact hospitalization costs, imaging utilization, and ultimately LOS [20–22]. Notably, these studies show the effect is largely achieved by replacing alternative imaging modalities (X-ray, Computed Tomography, etc.) with POCUS, not in triaging their use. It is imperative that time and resources be allocated to ensure adequate training and oversight, so that these benefits can be realized on a broader scale and with appropriate competency standards. Hospitalists (and hospital-based trainees) are uniquely poised to maximize the utility and benefits of POCUS, given the impact of POCUS on patient satisfaction, hospital throughput, and appropriate resource utilization [23].

Decision-making around the necessity for inpatient TTE remains complex in a POCUS-guided pathway and includes considerations such as image quality or limitations of the POCUS exam, clinical variables, and variability in practice patterns. However, we believe that utilizing POCUS for TTE triage addresses a gap that algorithm- or biomarker-based protocols have not been able to overcome. POCUS offers a technological solution to the increasing problems of declining physical exam and interpersonal skills that have been reported for at least the past 30 years, and the resultant tendency to overorder or inappropriately use advanced testing [24,25]. In this study, we took a broad approach to patient inclusion and found that more than half of patients identified by the POCUS-guided TTE triage pathway would be recommended for deferral or cancellation by both the POCUS hospitalist and at least one cardiologist. However, only about one-third of patients had full agreement between the POCUS hospitalist and both cardiologists. We expect that further tailoring the inclusion criteria could improve the rate of full agreement, though likely at the expense of deferral or cancellation rate. At our institution, we will continue to engage local champions and stakeholders to incorporate these preliminary findings into an operational change.

There are several limitations of this study to consider. First, as a single-institution study our results may not be broadly applicable to different practice environments owing to different patient populations, resource availability, and clinician skillsets. Second, clinician and patient survey response rates were 31% and 39%, respectively, which may have introduced sampling bias into our results. Third, we used a convenience sample approach to patient selection, which has potential for sampling and selection bias that may skew results, though notably there was no significant difference between the baseline and intervention patient demographics (Appendix 3). Lastly, we opted to utilize a tie-breaking strategy to best capture the majority opinion of participating cardiologists when resolving discrepancies in POCUS image interpretation rather than a “gold standard” comparison to TTE. We did this for two reasons: 1) 15% of patients included in the study ultimately had their TTE cancelled or not completed prior to discharge so comparison to TTE would not be possible, and 2) TTE has a reported inter-reader reliability of 8-13% [26], which we felt would introduce error from both differences in image quality/acquisition and interpretation. Therefore, while we were encouraged by these findings at our institution, we recommend considering these limitations and having discussions with local stakeholders before implementing them broadly.

## Conclusions

In this study, we demonstrated the feasibility of a POCUS-guided TTE triage protocol to reduce inappropriate inpatient TTE utilization. At our institution, both clinicians and patients were receptive to integrating POCUS into clinical care for certain clinical indications, with the caveat that trained clinicians perform and interpret those studies. When trained POCUS clinicians performed these exams within the recommended protocol, they completed exams in a timelier manner than TTE could be performed, and with moderate to very good reliability for specific cardiac findings. The proposed protocol has the potential to defer or cancel about half of inpatient TTEs for the specified cardiopulmonary conditions, with significant potential to expedite necessary TTEs, reduce TTE backlog, and ultimately reduce inpatient LOS for these patients.








