# Supplementary material for: Demonstrating Feasibility of Point of Care Ultrasound (POCUS)-Guided Inpatient Transthoracic Echo Triage Decision Pathway
Source: POCUS J. 2025 Apr 15;10(1):45–52. doi: 10.24908/pocusj.v10i01.17776 (PMC12057477; doi:10.24908/pocusj.v10i01.17776)
Supplement: Supplementary file 1 [file pocusj-10-01-17776-s001.pdf]

Appendix 1

Binary classification of qualitative point of care ultrasound (POCUS) findings by exam interpretation to facilitate pairwise analysis. LVSF, left ventricular systolic function; LV, left ventricular; RV, right ventricular.

Interpretation	0	1
**Pericardial effusion**	None<br>Trivial/physiologic<br>Small	Large
**LVSF**	Normal<br>Hyperdynamic	Reduced<br>Severely reduced
**LV size**	Normal	Small<br>Large
**RV size and function**	Normal	Abnormal
