# Supplementary material for: Demonstrating Feasibility of Point of Care Ultrasound (POCUS)-Guided Inpatient Transthoracic Echo Triage Decision Pathway
Source: POCUS J. 2025 Apr 15;10(1):45–52. doi: 10.24908/pocusj.v10i01.17776 (PMC12057477; doi:10.24908/pocusj.v10i01.17776)
Supplement: Supplementary file 2 [file pocusj-10-01-17776-s002.pdf]

Appendix 2

Demographics of all patients who underwent point of care ultrasound (POCUS) exam during the study period, comparing those who were not surveyed to those who consented to survey.

	**Not Surveyed (N=55)**	**Surveyed (N=35)**	**p-value (* = chi square, ^ = t-test)**
**Sex (N, %)**			**0.02***
Female	16 (29)	21 (60)	
Male	35 (64)	14 (40)	
**Age (Mean, SD)**	61.98 (15.18)	66.54 (12.91)	0.14^
**Body Mass Index (N, %)**			0.20^
< 18.5	1 (2)	1 (3)	
18.5 - 24.9	20 (36)	9 (26)	
25.0 - 29.9	14 (25)	8 (23)	
30.0 - 39.9	11 (20)	12 (34)	
> 40.0	5 (9)	5 (14)	
**Race (N, %)**			0.41*
Black or African American	20 (36)	19 (54.3)	
White	29 (53)	16 (45.7)	
Asian	1 (2)	0 (0)	
Decline to Answer	1 (2)	0 (0)	
**Health insurance (N, %)**			0.40*
Commercial	8 (15.7)	5 (14.3)	
Medicaid	11 (21.6)	4 (11.4)	
Medicare	29 (56.9)	25 (71.4)	
Other insurance or missing	3 (5.9)	1 (2.9)	
