# Supplementary material for: Demonstrating Feasibility of Point of Care Ultrasound (POCUS)-Guided Inpatient Transthoracic Echo Triage Decision Pathway
Source: POCUS J. 2025 Apr 15;10(1):45–52. doi: 10.24908/pocusj.v10i01.17776 (PMC12057477; doi:10.24908/pocusj.v10i01.17776)
Supplement: Supplementary file 3 [file pocusj-10-01-17776-s003.pdf]

Appendix 3

Demographics of all patients who were surveyed pre-intervention (“baseline”) vs. those who underwent point of care ultrasound (POCUS) exam during the study period (“intervention”).

	**Baseline (N=55)**	**Intervention (N=35)**	**p-value (* = chi square, ^ = t-test)**
**Sex**			0.771*
Female (N, %)	30 (54.5)	21 (60)	
Male (N, %)	25 (45.5)	14 (40)	
**Age** (Mean, SD)	63.75 (15.64)	66.43 (12.91)	0.379^
**Body Mass Index** (Mean, SD)			0.839^
Underweight (N, %)	3 (5)	1 (3)	
Healthy Weight (N, %)	14 (26)	9 (26)	
Overweight (N, %)	16 (29)	8 (23)	
Obese (N, %)	16 (29)	12 (34)	
Severe Obesity (N, %)	6 (11)	5 (14)	
**Race**			0.348*
African American (N, %)	23 (41.8)	19 (54.3)	
White (N, %)	32 (58.2)	16 (45.7)	
**Highest Level of Education**			0.180*
High school graduate or the equivalent (for example: GED) or less (N, %)	29 (52.8)	23 (65.7)	
Undergraduate studies or degree (N, %)	24 (43.6)	9 (25.7)	
Graduate degree	2 (3.6)	3 (8.6)	
**Health insurance**			0.726*
Commercial (N, %)	4 (7.3)	5 (14.3)	
Medicaid (N, %)	7 (12.7)	4 (11.4)	
Medicare (N, %)	43 (78.2)	25 (71.4)	
Other insurance or missing (N, %)	1 (1.8)	1 (2.9)	
