# Supplementary material for: Demonstrating Feasibility of Point of Care Ultrasound (POCUS)-Guided Inpatient Transthoracic Echo Triage Decision Pathway
Source: POCUS J. 2025 Apr 15;10(1):45–52. doi: 10.24908/pocusj.v10i01.17776 (PMC12057477; doi:10.24908/pocusj.v10i01.17776)
Supplement: Supplementary file 4 [file pocusj-10-01-17776-s004.pdf]

POCUS-Guided Inpatient TTE Triage

Can POCUS reduce unnecessary inpatient TTE utilization?

Purpose

Demonstrate feasibility of a POCUS-guided TTE triage protocol and estimate impact on inpatient TTE utilization

1. Are clinicians and patients receptive to POCUS?
2. Can POCUS be done quickly and reliably?
3. Can POCUS-guided triage help reduce low-value or inappropriate inpatient TTEs?

Study Protocol		Results
Clinicians surveyed **153 (31%)**	1 →	**Favored** Trained POCUS clinicians completing POCUS exams for TTE triage
POCUS exams performed for TTE triage **90**	2 →	**84%** Fair or good quality for interpretation
Patients surveyed post-POCUS exam **35 (39%)**	3 →	**4.37/5** Average rating of improved overall inpatient experience
TTE exams recommended for deferral or cancellation **59 (66%)**	4 →	**22 hours earlier** POCUS exams completed on average compared to TTE from TTE order
